# Is Dance Closer to Physical Activity or Spirituality? A Philosophical Exploration

**DOI:** 10.1007/s10943-021-01354-y

**Published:** 2021-08-09

**Authors:** Piotr Gronek, Joanna Gronek, Aleksandra Karpińska, Małgorzata Dobrzyńska, Paulina Wycichowska

**Affiliations:** 1 Faculty of Sport Sciences, Department of Dance and Gymnastics, Poznań University of Physical Education, Poznań, Poland; 2 Department of Tourism and Recreation, Poznań University of Physical Education, Poznań, Poland

**Keywords:** Philosophy, Soul, Recreation, Movement, Vitality

## Abstract

Dancing is inevitably associated with kinesthetics of the body, including movements, gestures, poses, jumps, turnings, transferring body weight, etcetera. Thus, dance is a manifestation of an amateur or the skilled behavior of a fully trained athletic performance. At the same time, dance is accompanied by emotions as an effect of expression, narration of choreography. Dance is also pre-planned and designed to produce numerous styles and techniques. It is a unique type of improvisation. However, in certain situations, styles and techniques that will be discussed below, the dancing body can bring the dancer closer to his/her emotionality and even spirituality. Thus, the aim of this philosophical exploration is to analyze the impact of spirituality on dance performance.

## Introduction

Dancing represents one of the best physical activities (PA) in terms of its health-promoting effects, and it is especially recommended for older adults (≥ 60 y) (Hopkins et al. 1990). It surpasses any fitness sport thanks to the widest impact spectrum to the body, including not only physical (endurance, muscular strength, flexibility), and psychological (cognition), but also social needs (ensuring the need of closeness, reducing of loneliness) (Gronek et al. 2021b; Rehfeld et al. 2017).

In detail, the positive effects of dance are elicited on cardiorespiratory fitness, psycho-motor skills (Koch et al. 2019), vascular aging (Seals et al. 2009), the nervous system (Verghese et al. 2003), as well as cognition, mood and the overall quality of life (McNeely et al. 2015). Among numerous definitions of dance, suggested, for example, by Horton-Fraleigh (1987), and Hanna (1983), the definition proposed by Copeland and Cohen assumes that dance is “any pattern, rhythmic movement in space and time” (Copeland & Cohen, 1983).

The medium of dance comprises five constituents, that is, the body, space, dynamics, action and relationships. Specifically the dimension classified as action when analyzed in terms of dynamics includes closing, contracting; opening, expanding; gesturing; jumping; locomotion; turning; transferring weight; and stillness. Additionally, the rhythm dimension includes the impactive (speeding up, accent at the end of the movement); impulsive (slowing down, accent at the beginning of the movement); rebounding (bouncing) and sustained rhythm (no accent, steady level of energy used). These structural elements are always present and articulated in dance regardless of its actual conjunction with music (Laland et al. 2016). Unfortunately, none of these definitions of dance mentions emotionality or spirituality, as they only analyze dance at the level of body movement/kinesthetics. And yet these aspects are also found in dance and provide it with an additional dimension.

However, spirituality vs. non-spirituality is not an unequivocal and explicit concept, in contrast to such juxtapositions as anger vs. emotional control, fear vs. courage, growth vs. degeneration, seed vs. fruit flesh, where these antithetical qualities seem distinct.

Spirituality can be defined as an intrinsic aspect of humanity, through which people seek ultimate meaning, purpose and transcendence and experience a relationship to self, family, others, community, society, nature and the significant or sacred (Puchalski et al. 2019). It is expressed through beliefs, values, traditions and practices (Puchalski et al. 2019). Moreover, spiritual distress may appear when there is an impaired ability to experience and integrate this meaning and the purpose in one’s life that can impact a patient’s health status (Hall & Powell, 2021). Thus, the aim of this philosophical exploration is to analyze the impact of spirituality on dance performance.

## Leisure Activity and the Comfort Zone

Among the different types and modes of leisure activities (reading, socializing, singing, or those more demanding and physically active such as walking, exercising, gardening, etc.), dancing is of particular interest because of combining bodily and emotional activity (Gronek et al. 2021a, b, c). Still it seems important what context is focused on and analyzed: healthy aging, well-being, sociality, individual development or other contexts (Gronek et al. 2019a, b, 2020). When studying healthy aging, Verghese et al. reported in the NEJM, based on a cohort of more than 450 subjects older than 75 years, that leisure activities such as playing board games, reading, playing musical instruments and dancing were associated with a reduced risk of dementia (Verghese et al. 2003). It is still surprising how poorly publicized and how underestimated such findings are.

Additionally, it has been shown that spirituality significantly improves the speed of recovery from certain illnesses, including neurologic disorders, cardiovascular disease, cancer, musculoskeletal disease and mental health problems (Ornish et al. 1990; Post et al. 2000; Pressman et al. 2013; Propst et al. 1992; Rąglewska & Domaszewska, 2020; Torosian & Biddle, 2003). However, it should be emphasized that although leisure activity is in general associated with pleasure, remaining in the comfort zone rather than engaging in processes that are more demanding, some modes of leisure activity could clearly extend beyond the comfort zone. The latter is observed in mountain climbing, extreme sports, community work for hospices and charity activities for people in need, etc. Is spirituality equivalent to remaining in the comfort zone? It probably depends how the comfort zone is defined, but the answer could be ambiguous and debatable.

## Motor Skills in Dance

The background of each style and technique is associated with appropriate motor skills as a function, which involves precise movement of muscles with the intent to perform a specific act. Main motor skills include strength, endurance and flexibility, since any specific dance technique may be learned only based on these skills (Gronek et al. 2019a, b; Włodarczyk et al. 2020).

Endurance training generally refers to developing and creating a higher level of efficacy of the aerobic metabolic systems that produces energy for working muscles (Chukhlantseva et al. 2020). The aim is to direct adaptations affecting the cardiovascular system, respiratory system and skeletal muscle tissue (Benzi et al. 1975; Clarke, 1973; Holloszy et al. 1977; Sharkey, 1970). In turn, resistance exercise is any form of exercise that causes the muscles to contract under external resistance, aiming to increase bone density and muscle hypertrophy (Borysławski et al. 2020). These are the basic activities that prepare the body of the athlete, including the dancer, to significant loads and high intensity of physical effort.

In some styles and dance techniques, including street dance or Afro dance, appropriate motor skills fulfill most of dancing requirements and tasks. Street dance is the best example in this respect. Street dance is a style of dance that has been developed outside dance studios in an open city space such as streets, dance parties, inner city housing projects, parks, school squares and nightlife venues.

Consequently, such dance styles represent a type of physical dance with an emphasis on acrobatic and gymnastic elements often used in improvisation; it is social in nature, encouraging interaction and contact with the audience and other dancers. These dances are part of the neighborhood culture.

Some physical similarity can be observed in Afro dance that is derived from the customs of individual ethnic groups; however, it comprises much less acrobatics and floor gymnastics. Dancers dance to live music produced by ballet musicians playing the djembe, dundun, balaphone and other instruments. This dance is divided into various techniques and rhythms depending on the tradition it refers to, such as catching fish, harvesting, child birth, wedding or funeral. Afro dance is very energetic, spacious and highly conditioned, demanding in terms of coordination and rhythm. Traditional Afro dance from West Africa allows participants to develop the dance technique in every respect, regardless of the style in which the dancer specializes. It helps to bring out strength and energy from every movement. It is possible to identify there elements of practically every subsequently developed style of dance: salsa, samba, hip-hop, jazz, modern, ballet and many more. Although it is used by residents daily to have fun, socialize and interact, it may hardly be considered as entirely devoid of spirituality. Especially when looking at happy and carefree children dancing Afro dance, it may not be excluded that this style at least potentially fulfills the hallmarks of spirituality.

## Between Physicality and Emotionality

Many dance styles and techniques in their performance combine physical and emotional dimensions, as evidenced by classical ballet, ballroom dances, modern dance, jazz dance, dance theater and Tanztheater. However, emotionality might range from a very simplified form aimed at impressing the audience or judges in ballroom dancing, to a deepened inner emotionality accompanied by a strong identification with the narrative, until reaching the state of the “tender narrator,” using the term popularized by the 2018 Nobel laureate Olga Tokarczuk. Such a deepened emotionality is characteristic of modern dance in general and probably especially of the “child” of modern dance, i.e., Tanztheater—derived from the expressionistic dance (nowadays a dance theater genre).

Among others, at least two conceptually different styles bring dancers closer to their emotionality: one of them is modern dance, while the other is butoh, a Japanese technique created by Hijikata (Stein, 1986). Butoh is an invitation to a journey into oneself for both the dancer and the audience. A butoh dancer focuses on every part of the body, on the back of the head, the forehead, elbows, and the toes. Standing on the stage and looking toward the last row of the audience, she/he fixes her/his eyes on the floor and beyond the audience. The inherent aesthetic core of butoh is the catharsis of the dancer, during which the dancer abandons the body and mind (Horton-Fraleigh, 2019).

Grotowski had a similar style of work in his Laboratory Theater, where the work was not only focused on mastering certain techniques and roles, but rather on “giving up the self.” The concept was for the actors to enter a trance state, striving for a state of kenosis, emptying self-denial and standing naked, not in the physical, but rather in the emotional, conscious and existential sense. Grotowski specified it as follows: We arm ourselves to hide, sincerity begins where we are vulnerable. In his view, honesty is impossible if we hide, we hide behind our clothes, to conceal ideas, signs, staging tricks and intellectual concepts. Instead, in the Laboratory Theater, they were replaced with gymnastics, shouting and chaos. If the method makes any sense, it is as a way of disarmament, not a technique. By disarmament, it is impossible to predict in advance what will happen and when it will happen, because it depends only on the existence of the person who completes the action (Grotowski, 2012 p. 99). Such an approach brings the dancer (actor) closer to catharsis and as a consequence invites also the audience to participate this process.

## When Dancing Comes Closer to Spirituality

A unique element of dance is improvisation of an activity in dance that does not follow a predetermined plan. The essence of improvisation is to completely focus on the current moment and on the activity performed at a given moment. The improvising dancer does not know what is going to happen next. Thus, one can speak of free or partially limited movement by determining the possibility of choice. Improvisation is used in almost all types of dance, from ethnic and folk, through hip-hop, to modern, post-modern, and contemporary dance.

For the spiritual dimension of dance, improvisation is important as an act of total surrender to the music or the inner impulses felt through the music. For a dancer, improvisation can also be a field of creative exploration.

When a dancer gives in to impulses and even pre-impulses, takes off technique, style and all learned roles and faces himself/herself internally, he/she allows the movement of consciousness in the structures of the imagination, realizing dreams, independent and distant. “It may be assumed that at this moment the dancer is deeply immersing himself/herself in the inner element of their psyche” (Gronek, 2016, pp. 49–52). It makes dance, in some circumstances, a viable psychotherapeutic treatment which is defined by The American Dance Therapy Association (ADTA) as “the psychotherapeutic use of movement to promote emotional, social, cognitive, and physical integration of the individual, for the purpose of improving health and well-being” (ADTA, 2020).

Art is generally seen as capable of uplifting and bringing people in touch with other dimensions (Shusterman, 2008). Dance is an exceptional activity, as it is created and experienced within and through the body: one is moving and feeling oneself moving at the same time (Kieft, 2014). Awareness of experiencing what one is expressing is the kind of somatic transformation emphasized by disciplines like yoga or breathing meditation. “It is an ultimate intimacy, a doing while being with oneself” (Sklar, 2014). What we embody in ourselves, we then bring “into our relationships with others and into the world” (Halprin, 2003).

Body-oriented therapies are based on this relationship between the body, movement, emotions and the psyche, including body-mind centering (Cohen, 1993; Halprin, 2003; Hartley, 1995), Gestalt therapy (Woldt & Toman, 2005) and Dance Movement Psychotherapy (Meekums, 2002; Penfield, 2001). Because of the multidimensionality of dance, a dancer recognizes himself or herself as a multidimensional agent moving through space, being aware of movement of his/her body parts, molecules and the connection to the life force and living organisms in general.

This experience leads to a capacity to create a relationship between the known and the unknown—the seen and the unseen dimensions of the world. The act of dancing becomes a possibility for feeling and expressing this two-way relationship, traffic or communication (Kieft, 2014). In this perspective, the body could be seen as a channel, through which “higher levels of consciousness” (Halprin, 2003) or one’s “spirit/soul” (Hume, 2007) work. Spiritual awareness is cultivated through simultaneous grasping of the physical, emotional and mental levels (Halprin, 2003; Worth & Poynor, 2004).

## Sacred Dances

In the context of dancing and spirituality especially sacred dances are of particular interest, as those are performed in rituals and religious ceremonies present in most religions. However, they are also found in the non-religious context where the sacred is closer to spirituality rather than traditionally understood religiousness. This latter category is traditionally divided into the following 5 subcategories: Hinduism, Christianity, Islam and Sufism, Syncretic, Spiritual and New Age.

Sacred dance is believed to have had several purposes, the most valuable being to honor the supernatural powers and to unite the dancer with such a supernatural power. This may be seen in the dances for Demeter and Persephone making the body suitable as a temporary dwelling-place for the deity, by dancing ecstatically to unconsciousness (Lawler, 1947). The other significant purposes were to honor the dead or prevent the ghost from leaving the grave, frighten off any evil spirits attracted by the corpse, or temporarily and invisibly bring the dead person back to join in the dance. At the end there were also minor purposes such as “to show off” before the supernatural powers.

Among the numerous Indian classical dances, such as Odissi, Kathak, Bharatanatyam, or Mohiniattam, some can even be traced to the Sanskrit text of Natya Shastra, probably written between 500 BCE and 500 CE (Khokar, 1984). Although they are traditional drama-dance expressions of religion, as related to Shaktism, Shaivism, Vaishnavism, pan-Hindu epics and the Vedic literature, they are performed either within the sanctum of a Hindu temple, or in its surroundings. These classical Indian dances seem to locate the core of the performance rather closer to a religious drama focusing on playing roles, the scenario and original costumes.

A slightly more complicated situation concerns Christianity. While some Christian traditions make use of liturgical or worship dance, it has long been controversial within at least some denominations. The early Christian church was in favor of dance, as in the second century AD the Acts of John, which states that “Grace danceth. I would pipe: dance ye all. The whole world on high hath part in our dancing” (Lihs, 2009). However, between 1685 and 1963 some 157 tracts against dance were written by monks and priests. There are only a few instances, including the Church of England, where Circle dances are used in its more meditative form. In southern Bulgaria and northern Greece, in the annual celebrations for Saint Helen and Saint Constantine, dancers perform the Anastenaria, a fire-walking ritual, that is the climax of three days of dancing, music, processions, and animal sacrifice (Danforth, 1989).

In Islam it should be noted that the truly hermitic lifestyle imposed on women had an impact on the distrustful attitude of medieval Islam toward dance and theater. However, dance is not unusual within Islam and in the tradition of the Mevlevi Order founded by Rumi, ecstatic Sufi whirling is practiced by devotees as a form of active meditation within the Sama (worship ceremony). Whirling Dervishes seem to be an example of the most profound spirituality in dance in general.

The Sufi Whirling Dances, Assyrian dances, and Eastern Sacred Dances became the starting point for the mystic and spiritual teacher George Gurdjieff, who collected or authored a series of sacred dances, known as the Gurdjieff movements. Dances were part of what he considered the Work of “self-observation” and “self-study” (Stanton, 1997). Although the main purpose termed Work proposed by Gurdjieff was associated with conscious living, an essential role in this system is played by movements consisting of exercises and dances.

The essential part in these exercises and movements is the “inner mathematics,” symbolics, rhythm, and music, composed mainly by Thomas de Hartmann. The concept of inner mathematics refers to choreography designed in such a way that, for example, 2 times rotation + 1 movement suspension + 1 jump + change of direction = sequence. A certain design of movements and sacred dances produces some traps for intellectual and emotional structures, which have to be focused directly on dancing, consequently enhancing the inner states and simultaneously distancing from external distractors. Additionally, including religious symbols and meanings in gestures and movements promotes deeper inner spirituality.

## Conclusions

The main difficulty concerning spirituality is that it is a deeply subjective inner state, inherently intangible and thus difficult to measure. This mean that speaking of “spirituality” we may not be absolutely certain what we are really talking about. Specifically one of the problems in the study of spirituality is related to the fuzzy semantics and terms such as spirit, soul, spirituality, which are not included in the same categories as inner space, consciousness, self-perception, intention, qualia or subjectivity (McSherry & Cash, 2004). Additionally, spirituality might be analyzed from at least four different views, i.e., evolution of spirituality (Demir, 2019), semantics (Chouiter & Annoni, 2018), anatomy (Li et al. 2019) and cognitive function (Hosseini et al. 2017). All the above has led to a suggestion that spirituality might be a multidimensional rather than a coherent phenomenon (Reed, 1992; Weathers et al. 2016).
